# Backbone phylogeny of *Salix* based on genome skimming data

**DOI:** 10.1016/j.pld.2024.09.004

**Published:** 2024-09-12

**Authors:** Kai-Yun Chen, Jin-Dan Wang, Rui-Qi Xiang, Xue-Dan Yang, Quan-Zheng Yun, Yuan Huang, Hang Sun, Jia-Hui Chen

**Affiliations:** a CAS Key Laboratory for Plant Diversity and Biogeography of East Asia, Kunming Institute of Botany, Chinese Academy of Sciences, Kunming 650201, Yunnan, PR China; b University of Chinese Academy of Sciences, Beijing 100049, PR China; c Beijing Ori-Gene Science and Technology Co Ltd, Beijing 102206, PR China; d School of Life Sciences, Yunnan Normal University, Kunming 650092, Yunnan, PR China

**Keywords:** *Salix*, Genome skimming, Phylogenomics, Orthologous coding sequencing, Subgeneric classification

## Abstract

The genus *Salix* is a common component of the Northern Hemisphere dendroflora with important ecological and economic value. However, taxonomy and systematics of *Salix* is extremely difficult and relationships between main lineages, especially deep phylogenies, remain largely unresolved. In this study, we used genome-skimming, plastome assembly, and single-copy orthologs (SCOs) from 66 *Salix* accessions, along with publicly available plastome and sequence read archive (SRA) datasets to obtain a robust backbone phylogeny of *Salix*, clarify relationships between its main lineages, and gain a more precise understanding of the origin and diversification of this species-rich genus. The plastome and SCO datasets resolved *Salix* into two robust clades, with plastome-based phylogenies lacking inner resolution and SCO offering fully resolved phylogenies. Our results support the classification of *Salix* into five subgenera: *Salix*, *Urbaniana*, *Triandrae*, *Longifoliae* and *Vetrix*. We observed a significant acceleration in the diversification rate within the *Chamaetia-Vetrix* clade, while *Salix* exhibited increased rates of diversification spanning from the early Oligocene to the late Miocene. These changes coincided with contemporaneous tectonic and climate change events. Our results provide a foundation for future systematic and evolutionary studies of *Salix*. Additionally, we showed that genome skimming data is an efficient, rapid, and reliable approach for obtaining extensive genomic data for phylogenomic studies, enabling the comprehensive elucidation of *Salix* relationships.

## Highlights

• ∼25% of *Salix* species were sampled for plastome and nuclear single copy orthologs (SCO), covering all main *Salix* lineages.
• Plastome and SCO-based phylogenies of *Salix* are incongruent.
• The SCO data matrix with ca. ∼3.7 Mb sites provide a robust backbone phylogeny for *Salix*.
• The rate of diversification within the *Chamaetia-Vetrix* clade accelerated from the early Oligocene to the late Miocene.

## 1 Introduction

*Salix*, commonly known as willows, is the largest genus in the family Salicaceae s.str. with approximately 400–520 species (Fang et al., 1999; Skvortsov, 1999; Ohashi, 2001; Argus et al., 2010). Willows are economically important because of their application in soil engineering, landscape gardening, ornamental use, wind prevention, bioremediation, and biomass production (Fang et al., 1999; Skvortsov, 1999; Karp and Shield, 2008; Argus et al., 2010). *Salix* are also of great interest to biologists as willows are one of the largest dendroflora genera of the Northern Hemisphere, with members dominating woody plants in well-watered habitats, such as natural wetlands, river valleys, stream banks, and lake shores. Despite the significant ecological and economic value of willows, the genus *Salix* poses a challenge for taxonomists and systematists. Morphological classification is hindered by a number of factors, including extensive variation in phenotypes within species, frequent natural hybridization, sex differentiation in plants, and differences in developmental stages between flowers and leaves (Rechinger, 1992; Skvortsov, 1999; Argus et al., 2010; Percy et al., 2014).

Molecular phylogenetic studies have confirmed that *Salix* is monophyletic and proposed that the genus consists of two main lineages, here referred to as Clade 1 and Clade 2 (Azuma et al., 2000; Chen et al., 2010; Lauron-Moreau et al., 2015; Wu et al., 2015; Zhang et al., 2018b; Gulyaev et al., 2022). Clade 1 contains most species of subg. *Salix*. Clade 2 comprises the subg. *Chamaetia-Vetrix* clade, along with some subg. *Salix* species (*Salix arbutifolia, S*. *cardiophylla* and sect. *Triandrae*) (hereafter referred to as Clade 2). However, these phylogenetic relationships have recently been challenged. Most studies have been conducted using a limited number of molecular markers (Leskinen and Alstrom-Rapaport, 1999; Azuma et al., 2000; Chen et al., 2010; Lauron-Moreau et al., 2015; Wu et al., 2015). Only those based on plastid sequences have been able to confirm the aforementioned results (Azuma et al., 2000; Chen et al., 2010; Lauron-Moreau et al., 2015; Wu et al., 2015; Zhang et al., 2018b; Gulyaev et al., 2022). Conversely, phylogenetic studies that rely on a small number of nuclear sequences (such as ITS and ETS) have failed to resolve the two major lineages of *Salix* (Leskinen and Alstrom-Rapaport, 1999; Lauron-Moreau et al., 2015; Wu et al., 2015).

Studies have indicated that the relationships observed in deep branches of *Salix* (Clade 1 and Clade 2) and in the largest and most species-rich clade (*Chamaetia-Vetrix* clade) are inconsistent. For instance, phylogenetic studies of *Salix* based on restriction-site associated DNA sequencing (RADseq) data found that the *Chamaetia-Vetrix* clade consists of two robust clades (He et al., 2021; Marinček et al., 2024). A study based on single nucleotide polymorphism (SNP) sites did not recognize Clade 1 and divided the *Chamaetia-Vetrix* clade into two robust clades (Gulyaev et al., 2022). A study based on 787 genes also failed to recognize Clade 1 but did not divide the *Chamaetia-Vetrix* clade into two (Sanderson et al., 2023). One limitation of these studies is that they sampled a relatively limited number of species within this clade. Consequently, the relationships within the *Chamaetia-Vetrix* clade remain largely unresolved and require further investigation with more comprehensive sampling.

Plastomes of *Salix* have proven to be highly conserved and useless in resolving relationships of this large clade (Huang et al., 2017; Zhang et al., 2018b; Wagner et al., 2021). Therefore, to elucidate the relationships of the main lineages of *Salix* as well as the *Chamaetia-Vetrix* clade, phylogenomic analysis using a large number of nucleotide sites with more comprehensive sampling is needed. Genome skimming offers a cost-effective, rapid, and reliable method for obtaining genomic sequence data through high-throughput sequencing (HTS). It can be used to assemble the plastome and, occasionally, even mitochondrial genomes (Nevill et al., 2020; Liu et al., 2021). The HybPiper pipeline is capable of retrieving single-copy orthologs (SCOs) of interest by assigning HTS reads to target genes (i.e., SCOs). The sequenced reads can subsequently be separated and assembled separately (Johnson et al., 2016).

In this study, we sequenced and assembled genome-skimming plastomes and retrieved SCOs from 66 *Salix* accessions. Additionally, plastomes and publicly available sequence read archive (SRA) datasets were incorporated, resulting in a data matrix of 67,901 bp and 3,654,192 bp for the plastome and SCO datasets with 173 and 191 samples from 124 to 128 *Salix* species, respectively (Table S1). This is the first phylogenomic study of *Salix* using large numbers of DNA sites from plastome and nuclear sequences with more samples than previous studies, representing all *Salix* lineages identified in previous phylogenetic studies, and was used to estimate the most comprehensive and robust backbone phylogeny to date. We then conducted divergence time estimation and diversification rate analyses. We aimed to obtain a robust backbone phylogeny of *Salix*, clarification relationships between main lineages, and to gain a more precise understanding of the origin and diversification of this species-rich genus.

## 2 Materials and methods

### 2.1 Materials and genome skim-sequencing

We collected 66 samples from 60 *Salix* species that belong to three subgenera (Table S2). Fresh leaves were collected and dried using silica gel for subsequent DNA isolation. DNA isolation was carried out using a modified cetyltrimethylammonium bromide (CTAB) method (Doyle and Doyle, 1987). Purified DNA (5 μg) was fragmented, and short-insert libraries were constructed following instructions provided by the manufacturer (Illumina, Inc., San Diego, CA, USA). DNA from the various individuals was indexed by tag and pooled together in one lane of an Illumina HiSeq X Ten platform, producing 2 × 150 bp paired-end reads, resulting in approximately 4.5 Gb of raw data per sample (Table S1).

### 2.2 Plastome and SCO assembly

To obtain nuclear genome sequences for phylogenomic analysis, genome skimming data were generated from 66 *Salix* accessions sequenced in this study and 103 *Salix* SRA data downloaded from the NCBI. Raw Illumina data from sequenced *Salix* samples and SRA data were subjected to sequence quality filtration using Trimmomatic v.0.39 (Bolger et al., 2014) with default parameters. The filtered reads were retrieved and used to assemble plastomes using NOVOPlasty v.4.3.1 (Dierckxsens et al., 2017) with *Salix oreinoma* as the reference genome (GenBank accession number: NC_035743). Assembled plastomes were annotated using GeSeq (Tillich et al., 2017). Newly generated plastomes of the 77 *Salix* samples in this study were submitted to the China National Center for Bioinformation (CNCB, https://www.cncb.ac.cn/) (Table S3).

To identify orthologous sequences, six Salicaceae species (i.e., *Populus deltoides*, *P. trichocarpa*, *Salix* *arbutifolia*, *S*. *brachista*, *S*. *dunnii*, and *S*. *purpurea*) with publicly available high-quality whole-genome assemblies were used (Tables S1 and S2). We identified 6238 SCOs across six species using OrthoFinder v.2.5.4 (Emms and Kelly, 2019). Subsequently, HybPiper v.2.1.6 (Johnson et al., 2016) was employed, which initiates with the aforementioned filtered clean sequencing reads and maps them to the most closely related target protein-coding genes (CDS) (i.e., SCO) via BlastX (Li and Durbin, 2009). The reads were then distributed and assembled into contigs independently using SPAdes v.3.15.5 (Bankevich et al., 2012) with a coverage cutoff value of 5, and Exonerate v.2.2.0 (Slater and Birney, 2005) to align the assembled contigs to target sequences and determine exon and intron boundaries. The SCO sequences were then retrieved (“hybpiper retrieve_sequences”), and assembly statistics and recovery efficiency were also obtained using the “hybpiper stats” and “hybpiper recovery_heatmap” functions.

### 2.3 Phylogenetic tree construction

For phylogenetic inference purposes based on the plastome, we compiled a dataset of our 65 newly generated *Salix* plastomes, 114 publicly available *Salix* plastomes from NCBI (https://www.ncbi.nlm.nih.gov/), 12 newly assembled *Salix* plastomes based on publicly available *Salix* SRA data in NCBI, and three *Populus* plastomes as outgroups (Tables S1 and S3). The plastomes obtained from NCBI were reannotated using GeSeq (Tillich et al., 2017). Phylogenetic reconstruction was performed using 75 CDSs that were present in at least 193 samples with a total alignment length of 67,901 nucleotide sites (Table S4). The CDSs were aligned using MAFFT v.7.475 software (Katoh and Standley, 2016). Additionally, we evaluated the best-fit model of evolution for each CDS with the minimum Bayesian information criterion score computed by ModelFinder (Kalyaanamoorthy et al., 2017) (Table S4). Phylogenetic inference was then conducted by the maximum likelihood (ML) method using IQ-TREE v.2 (Minh et al., 2020), and parameters were estimated separately for each CDS using an edge-linked proportional partition model that accounts for different evolutionary speeds and separate substitution models and separate rates across sites for partitions (Chernomor et al., 2016). The ML tree was inferred 20 times independently, and the ML tree with the highest log-likelihood was ultimately selected. Support values were calculated using IQ-TREE from 5000 replicates with the SH-aLRT (SH-like approximate likelihood ratio test) (Guindon et al., 2010) and UFBoot (ultrafast bootstrap) (Hoang et al., 2018) methods.

SCOs were obtained for phylogenetic analysis from two *Populus* and 173 *Salix* accessions. Assembled sequences with non-missing lengths less than 250 bp were first culled, and the sequences within each retrieved SCO were subsequently aligned using MAFFT v.7.475 (Nakamura et al., 2018) with the “localpair -maxiterate1000” settings. Due to the varying sequencing depths in the genome skimming data, we applied trimAL v.1.2 (Capella-Gutierrez et al., 2009) to trim the alignment of each SCO. The trimming process removed poorly aligned columns, which contained more than 25% gaps in the sequences or had a similarity score lower than 0.001 (“-gt 0.75 -st 0.001”). Additionally, we removed sequences that had a length less than 250 bp since the short sequences in each alignment offered limited informative sites. Furthermore, to mitigate the impact of missing data, orthologous alignments were maintained only if they included at least 95% (166) of the samples. We filtered out 1449 SCO alignments for subsequent analysis. The cumulative length of these alignments was 3,654,129 bp, within which 1,143,079 variable sites (31.3%) and 725,118 (19.8%) parsimony informative sites were found (Table S1). To search for the best-scoring ML tree based on the GTR + G model, we used RAxML-NG (v.0.9.0) and ten random and parsimony-based starting trees. Following the same model specification, we performed 300 nonparametric bootstrap replicates using the best-scoring ML tree as the starting tree. The reliability of the best-scoring ML tree was evaluated using transfer bootstrap expectation (TBE). TBE was suggested by Lemoine et al. (2018). Each branch of the ML tree was compared with its respective closest branch in the bootstrap replicate tree. In large trees with numerous taxa, the standard bootstrap metric (Felsenstein’s bootstrap, FBP) usually yields low support, especially for deep branches, due to the rarity of bipartitions that exactly match those in the best-scoring ML tree. Therefore, TBE is a more effective method of recovering support in large trees (Kozlov and Stamatakis, 2020). The SH-aLRT support was assigned to the best-scoring ML tree using IQ-TREE v.2 with 10,000 replicates. We also estimated ML gene trees for each SCO alignment using RAxML v.8.2.4 with a GTRGAMMA model and 100 bootstrap replicates to evaluate clade support. Newick Utilities (https://github.com/tjunier/newick_utils) were utilized to collapse branches with less than 10% bootstrap support within each gene tree. Subsequently, we proceeded to construct a species tree through the use of the coalescent-based method ASTRAL-III v.5.7.1 (Zhang et al., 2018a). Local posterior probabilities (Sayyari and Mirarab, 2016) were utilized to assess clade support.

To assess the topological consistency of the SCO-based phylogenetic tree, we constructed a ML tree using VeryFastTree v.4.0.3 (Piñeiro et al., 2020) under the GTRGAMMA model. The analysis utilized a concatenated matrix of filtered and trimmed 6238 SCOs, spanning a length of 9,420,253 bp. A total of 5,692,996 sites (60.4%) were variable and 4,081,058 sites (43.3%) were parsimony informative (Table S1). Chen et al. (2019) previously reported 390 SCOs shared by 11 Salicaceae species (SCO-11sp), 253 of which were identified within our 6238 SCOs. These were identified as gene names of *Salix* *brachista*, which is the only species with the same version of genome used for identifying SCOs by Chen et al. (2019) and our current study. The cumulative length of these selected SCOs was 378,006 bp, with 116,953 (30.9%) variable sites and 73,687 (19.5%) parsimony informative sites (Table S1). An ML tree was therefore reconstructed employing an edge-linked partition model in IQ-TREE following aforementioned methods.

### 2.4 Divergence time estimation

Divergence times were estimated using the ML tree constructed based on the concatenated SCO dataset under a relaxed molecular clock model by MCMCTree (Yang, 2007). The approximate likelihood method, along with an independent substitution rate (clock = 2) and a GTR (model = 7) substitution model, were employed. Samples were drawn every 10 iterations until 10^7^ iterations were completed; afterwards, we ran 1.1 × 10^8^ iterations and discarded 10^7^ iterations as the burn-in. To ensure convergence to the stationary distribution, each analysis was performed twice, and the results were subsequently compared. The root age of the tree was calibrated to 48–52 million years ago (Ma) based on reliable fossils of *Populus tidwellii* (most likely a member of the stem lineage leading to *Populus* and *Salix*) (Manchester et al., 2006) and *Pseudosalix* (an early divergent extinct member of the stem lineage of *Populus* and *Salix*) (Boucher et al., 2003), along with estimations by Dai et al. (2014). The crown age of the subg. *Vetrix* clade was calibrated to 23 Ma based on the earliest reliable *Salix* fossils found in Alaska, which belong to subg. *Vetrix* and had both catkins and leaves from the late Oligocene (Collinson, 1992). We performed two independent Markov chain Monte Carlo (MCMC) runs for comparison of the results to check convergence of the stationary distribution. Tracer v.1.7.1 (https://beast.community/tracer) was used to determine the resulting effective sample size (ESS) for each parameter, with all parameters having an ESS > 200.

### 2.5 Diversification rate analysis

To improve our understanding of the dynamics of lineage diversification in *Salix*, we examined speciation (*λ*), extinction (*μ*), and net diversification (*r*) rates through evolutionary time and across the *Salix* phylogeny using BAMM v.2.5.0 (Rabosky, 2014) and the R package BAMMtools to generate Bayesian inferences of diversification rates based on time-calibrated phylogenies obtained from our MCMCtree analysis. We used clade-specific sampling fractions that specified for main clades identified in *Salix*, i.e., *Salix*, *Urbanianae*, *Triandrae, Longifoliae* and *Chamaetia-Vetrix*; detailed sampling fractions are listed in Table S5*.* We therefore accounted for incomplete sampling. Speciation and extinction priors were determined empirically using the setBAMMpriors function. The expected number of regime shifts was set to 1 by means of a geometric prior, as advised in the BAMM documentation. All BAMM Markov chain Monte Carlo analyses were conducted for 100 million generations, with a sampling frequency of 1/1000. We evaluated convergence by visually examining likelihood trace plots and computing the effective sample size while discarding the first 25% of the run as burn-in. The rate-through-time plots of speciation, extinction and net diversification over time were summarized and plotted using the *PlotRateThroughTime* function of BAMMtools.

## 3 Results

### 3.1 Plastome-based phylogenetics

The *Salix* plastomes we analyzed included 191 accessions from 128 species and 44 sections of all seven *Salix* subgenera. Of these, 77 plastomes were newly generated for this study and the remaining 114 were downloaded from NCBI (Table S1). The dataset included 75 CDSs present in at least 193 samples with an alignment length of 67,901 sites, of which 1662 (2.45%) were parsimony informative (Tables S1 and S4).

**Fig. 1:**
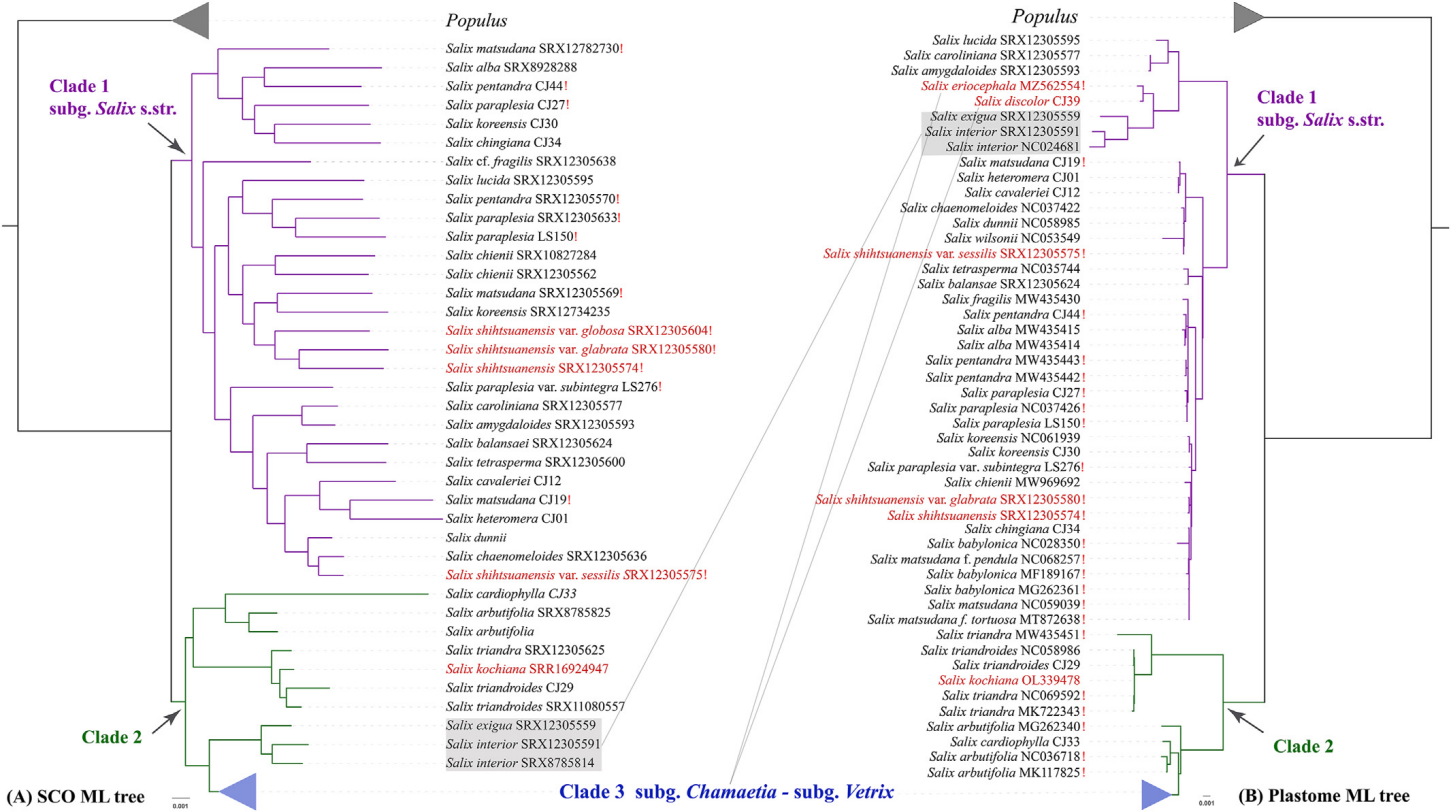
Maximum likelihood tree of *Salix* inferred from the concatenated SCO dataset (A) and the plastome CDS dataset (B). Grey lines connect conflicting taxa between the two phylogenetic trees, and red exclamation points indicate accessions of species that are not monophyletic. The branch supports of the main clades (clades 1, 2, 3) are SH-aLRT/UFBoot = 100/100 for the palstome-based ML tree and transfer bootstrap expectation (TBE) of 1 for the SCO-based ML tree. Branch support values for all clades are shown detail in Figs. S1 and S3.

The findings of phylogenetic analysis based on the plastome data are consistent with previous studies that also used plastome (Wagner et al., 2021) or a small number of plastid sequences (Chen et al., 2010; Wu et al., 2015). This confirms the monophyly of *Salix* and the existence of two major clades of *Salix* (Chen et al., 2010; Wu et al., 2015; Zhang et al., 2018b). Clade 1 comprises species from the subg. *Salix*, with reciprocal monophyly between New and Old World species in this clade, with five exceptions from the subg. *Vetrix*, namely, *Salix eriocephala*, *S*. *discolor*, *S*. *shihtsuanensis* and its three varieties (Figs. 1B and S1). Clade 2 comprises two clades. The first clade includes the two species of sect. *Triandrae* and *Salix kochiana* (subg. *Vetrix*, sect. *Caesiae*). The other clade consists of two sister clades, namely, the *Chamaetia-Vetrix* clade and the clade consisting of subg. *Pleuradenia* (*S. cardiophylla*) and two accessions of subg. *Chosenia* (*S. arbutifolia*). One accession of *S. arbutifolia* is a sister to other samples of this clade (as shown in Figs. 1 and S1). The resolution within the *Chamaetia-Vetrix* clade was low, as indicated by the presence of short branches and low or no SH-aLRT or UFBoot support values for most branches (Fig. S1). This is reasonable when considering the highly conserved structure and content of plastomes in Salicaceae s.str., particularly in *Salix* species (Huang et al., 2017; Zhang et al., 2018b). Our findings demonstrate that resolving phylogenetic and systematic issues within the highly diverse *Chamaetia-Vetrix* clade requires the use of a large number of informative DNA sites, such as those obtained through techniques such as whole-genome resequencing or genome skimming. It is essential to obtain and utilize such data to achieve a comprehensive understanding of the evolutionary history of this clade.

### 3.2 Genome skimming-based phylogenomics

**Fig. 2:**
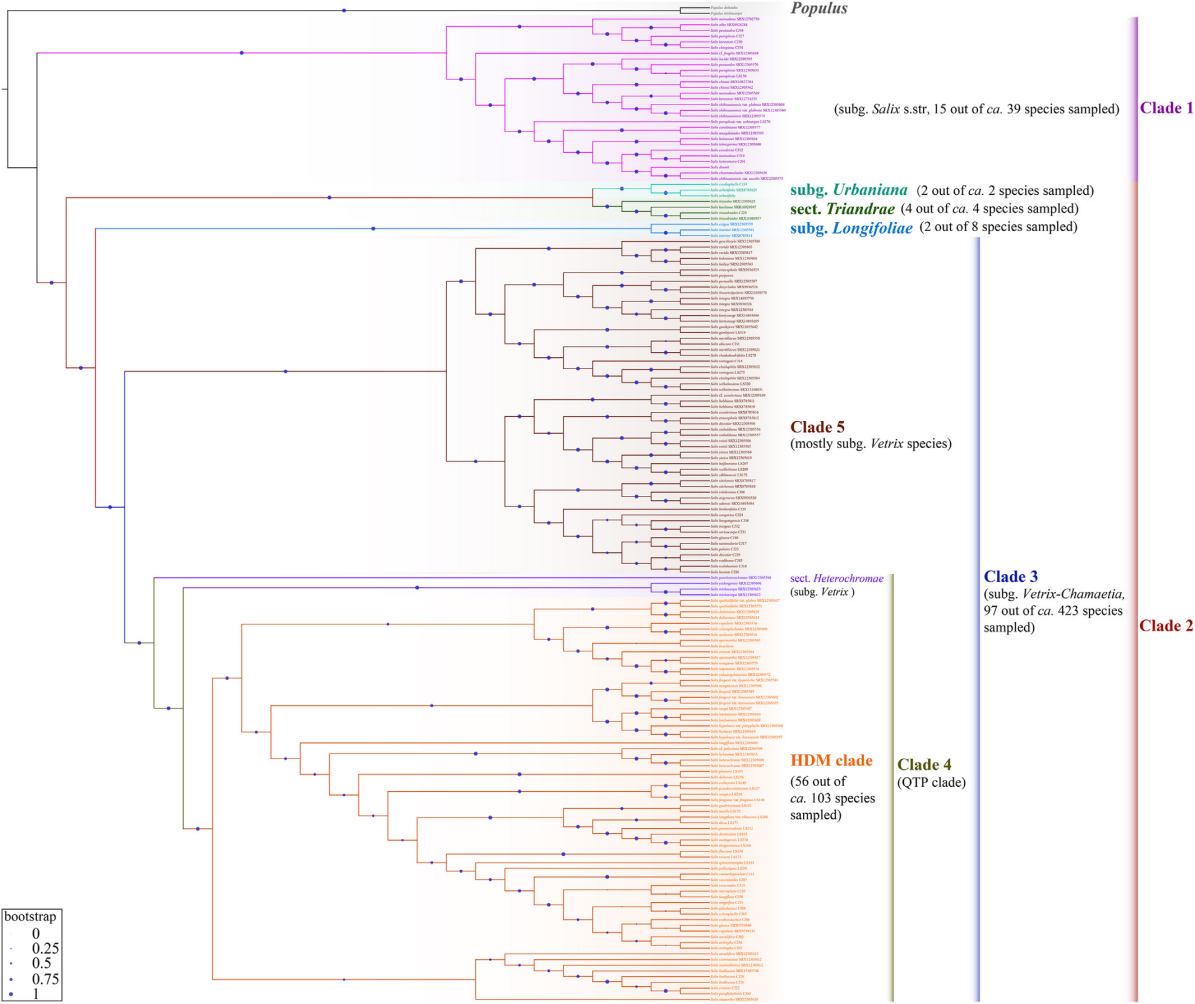
Cladogram of concatenation-based species tree inferred by RAxML-NG of 1449 SCOs.

A total of 173 *Salix* accessions were obtained, comprising 124 species from 40 sections and seven subgenera, according to the systems of Argus et al. (2010) and Ohashi (2001) (Tables S1 and S2). Fig. S2 illustrates the recovery efficiency of each of the 6238 SCOs shared by the six Salicaceae species. The quality of the SCO retrieved was fairly high. The length and depth of the assembled SCOs are listed in Tables S6 and S7, respectively. We filtered out 1449 high-quality SCOs for phylogenetic analysis. The total data matrix was 3,654,192 bp in length, with 725,118 (19.8%) sites being parsimony informative (Tables S1 and S8). Subsequently, we employed RAxML-NG to construct a best-scoring maximum likelihood (ML) tree with the GTR + G model utilizing the concatenation alignment of all 1449 SCOs (Fig. 1, Fig. 2, S3 and S4). We constructed a species tree through Astral based on each of the 1449 SCO gene trees (Fig. S5). ML trees based on 6238 SCO and 253 SCO-11sp datasets, which have alignment lengths of 9,420,253 bp and 378,006 bp, respectively (Tables S1 and S9), yield similar topologies to that of 1449 SCO (Figs. S6 and S7). The SCO-based ML trees and the Astral species tree revealed consistent relationships for the main *Salix* lineages, with the exception of a few conflicting relationships (Fig. S8). This demonstrates the robustness of the phylogenetic reconstruction based on the SCO dataset. Genome skimming-based phylogenetic trees, like the plastome-based ML tree, revealed two robust clades, clade 1 and clade 2. However, the structure of clade 2 differs somewhat: the *Chamaetia-Vetrix* clade (clade 3) is sister to a clade comprising two species of subg. *Longifoliae* (clustered in clade 1 in the plastome-based ML tree). Moreover, they are sister to a clade that includes two sister clades, subg. *Chosenia-Pleuradenia* clade and sect. *Triandrae-S. kochiana* clade.

Genome skimming-based phylogenetic analysis provided good resolution in the species-rich *Chamaetia-Vetrix* clade compared to plastome-based phylogenetic inference. Clade 3 was further divided into two clades, while clade 4 comprised species of subg. *Chamaetia* with distribution areas within the Qinghai-Tibet Plateau (QTP). This included three exceptions, namely, *Salix sphaeronymphe* of subg. *Salix*, *Salix yuhuangshanensis* and *S*. *hylonoma* of subg. *Vetrix*, as shown in Fig. S5. In some cases, there were four exceptions, including *S*. *glauca* (ERX5334560), as shown in Figs. 2, S3 and S4. Furthermore, the species distributed in the Hengduan Mountains (HDM) and adjacent regions in clade 4 formed a robust clade, except for one (*S. yuhuangshanensis*) (Fig. S5) or two (plus an accession of *S. glauca*) (Figs. 2, S3 and S4). In the Astral species tree, Clade 5 primarily comprises species of subg. *Vetrix* and some members of subg. *Chamaetia*: four arctic-alpine dwarf *Salix* species belonging to subg. *Chamaetia* (*S. glauca*, *S*. *polaris*, *S*. *nummularia*, and *S*. *berberifolia*), three subg. *Salix* species distributed in Northwest China (*S*. *songarica*, *S*. *bangongensis*, and *S*. *sericocarpa*), and two species of subg. *Chamaetia* sect. *Psilostigmatae* (*S*. *balfouriana* LS207, *S*. *sikkimensis* LS153), *S*. *wolohoensis* (subg. *Chamaetia* sect. *Eriocladae*) (Figs. 2 and S3–S5), and *Salix paraheterochroma* (SRX12305598) (Fig. S5). The ML tree based on concatenation shows that clade 5 consists of two clades, with samples belonging to subg. *Chamaetia* in clade 5 (*S. berberifolia, S. glauca, S. polaris* and *S. nummularia*) grouped in one clade (Figs. 2 and S3–S4). In the Astral species tree, however, these subg. *Chamaetia* taxa were scattered among the two sister clades of clade 5 (Fig. S5).

### *3.3* *Divergence times of the main clades of* Salix

**Fig. 3:**
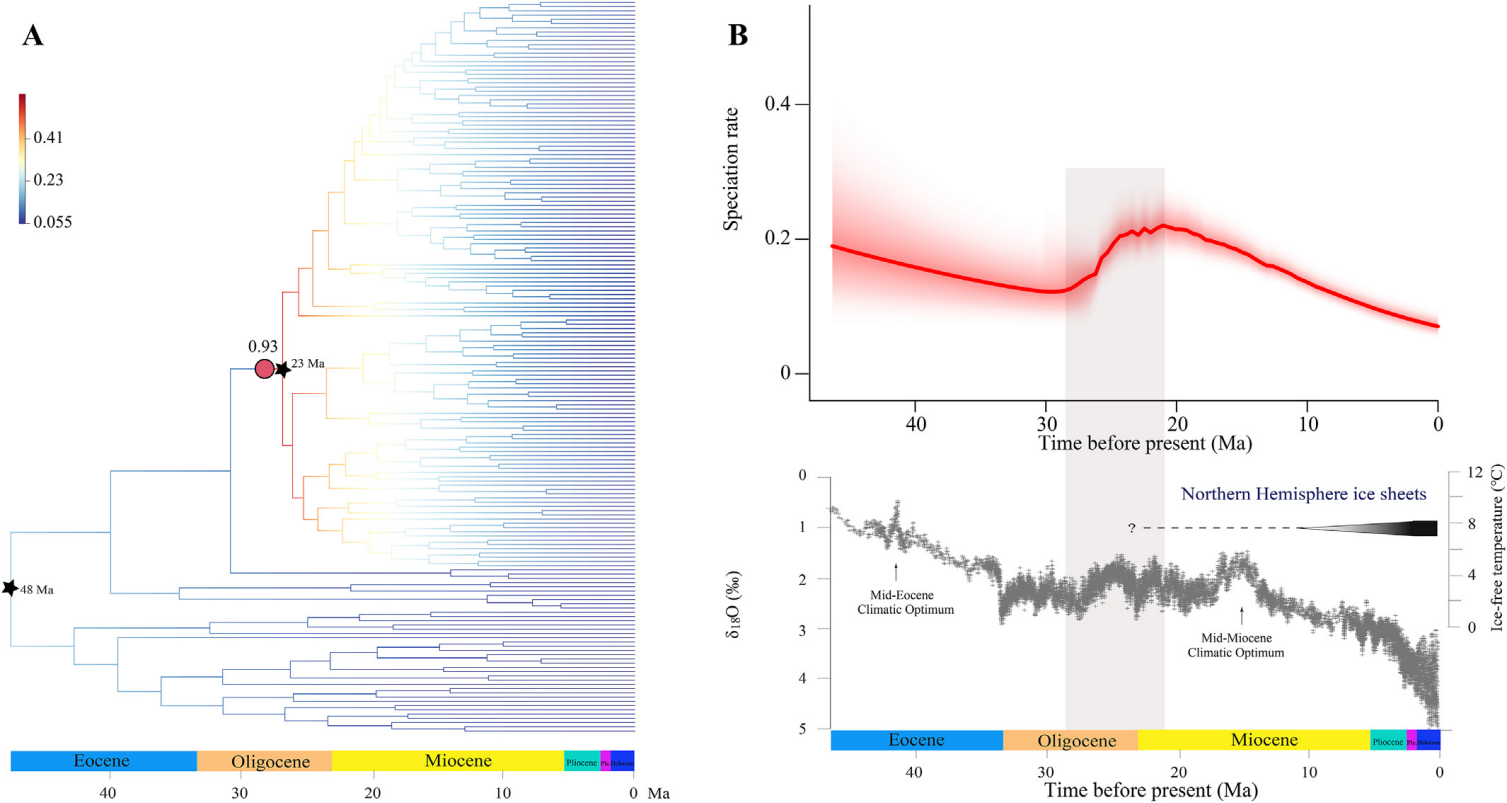
(A), Net diversification through lineages and the best-fit rate shift configuration for *Salix*. Colors of branches show the mean diversification rate (species/million years) from BAMM. Red circles indicate node of rate shift with probability. Nodes used for calibration and the corresponding calibrated time are marked with black pentagram. (B), Net diversification rate through time for *Salix*, and comparison with global climate change over the last 46 million years with the ice-free temperature scale on the right axis and the left δ^18^O temperature scale on the left axis (modified from Zachos et al., 2008).

We estimated the divergence time of the primary *Salix* lineages using an ML tree generated via concatenation. Our results show that subg. *Salix* (clade 1), *Chamaetia-Vetrix* (clade 3), QTP (clade 4) and HDM clades diverged at 46.34 Ma (95% HPD: 39.77–50.8 Ma), 41.64 Ma (95% HPD: 34.51–47.19 Ma), 26.15 Ma (95% HPD: 23.31–27.95 Ma), 24.96 (95% HPD: 21.97–27.12 Ma), and 22.73 Ma (95% HPD: 19.38–25.23 Ma), respectively (Fig. S6). The divergence times of the other main clades of *Salix*, as estimated using SCO datasets, are shown in Fig. S9. Our findings are generally consistent with those of previous studies (Wu et al., 2015; He et al., 2021). We identified a significant acceleration in the diversification rate of the *Chamaetia-Vetrix* clade, while *Salix* exhibited increased diversification rates from the early Oligocene (around the Chattian stage) through the late Miocene (approximately the Aquitanian stage) (Fig. 3).

## 4 Discussion

### 4.1 Genome skimming data are powerful for resolving Salix *phylogenies*

In this study, we successfully retrieved thousands of orthologous sequences between six Salicaceae species, which contain a substantial number of informative nucleotide sites for phylogenomic inference, using HybPiper. We obtained nearly fully resolved phylogenies for the notoriously challenging plant taxon *Salix*. Traditional phylogenetic analysis based on limited DNA markers failed to resolve these relationships, and even plastome-based phylogenomic studies were unsuccessful due to the highly conserved nature of the *Salix* plastome. Genome skimming utilizes high-throughput next-generation sequencing technology to generate low-pass shallow sequencing data (approximately 5% coverage), as demonstrated in our study, with a data size as low as 3.29 Gb (SRX9739132, *Salix cupularis*). Compared with other sequencing technologies, such as RADseq, RNAseq, and whole-genome resequencing methods, genome skimming is a cost-effective, rapid, and reliable approach that can be employed to uncover conserved orthologous sequences and, in some cases, even plastomes or mitochondrial genomes for phylogenomic studies (Johnson et al., 2016; Nevill et al., 2020). Moreover, DNA sequences are linear strings of nucleic acids consisting of two distinct types of characters: constant and variable. Once aligned, these sequences can be analyzed using model-based or coalescent-based species-tree estimation methods (Leaché and Oaks, 2017; Kapli et al., 2020). This approach confers an advantage to sequence-based phylogenetic analysis over single nucleotide polymorphism (SNP) data-based approaches due to the inherent acquisition bias in SNP data, which exclusively comprises variable characters. Such bias can lead to significant overestimation of evolutionary rate inaccuracies in topologies, branching times, and support values (Leache et al., 2015). A significant number of plant samples have undergone genome skimming sequencing, primarily for the purpose of plastome assembly. Consequently, there is a vast repository of publicly available genome skimming data. Our study emphasizes the untapped potential of these sequences as hidden resources that can be further utilized to retrieve numerous orthologous sequences within a plant family. These sequences can contribute to the resolving of taxonomic relationships among plant taxa, particularly for those that are systematically challenging. Furthermore, these findings provide a comprehensive evolutionary history beyond phylogenetic studies that rely solely on uniparentally inherited plastid and mitochondrial genomes.

### 4.2 Nonmonophyly of multiple species

Nonmonophyly was detected in numerous species across both subg. *Salix* and *Vetrix* clades (Fig. 1, Fig. 2, S1 and S3–S5), in contrast to two previous phylogenetic studies that used RADseq and sampled fewer *Salix* species (15 and 35, respectively) (Wagner et al., 2018, 2020). These studies revealed that all the accessions of each species formed monophyletic clades. Previous research based on RADseq revealed that three accessions of *S. glauca* were nonmonophyletic and scattered across three lineages (Marinček et al., 2024). The authors proposed that this nonmonophyly may have resulted from a possible independent allopolyploid origin or a hybridization event. Similarly, two accessions of *S. glauca* in our trees were nonmonophyletic. Within our trees, the three accessions of *S*. *integra* did not form a monophyletic clade but instead grouped in a clade with *S*. *koriyanagi*. However, four accessions of *S. integra* were previously shown to be monophyletic (Wagner et al., 2020). However, this study did not sample *S. koriyanagi.* The distribution areas of these two species overlap in the Korean Peninsula and Japan; therefore, there is a possibility of natural hybridization or introgression between these two species. Our study’s samples differ from those of Wagner et al. (2020) because RADseq data cannot be used to retrieve orthologous nuclear sequences and therefore could not be included in our study. In a previous study by Gulyaev et al. (2022), two accessions of *S*. *myrtillacea* were found to form a clade in a phylogenetic tree based on SNP data. However, in our sequence-based phylogenies, which included more species, these same samples were not monophyletic and instead formed a clade with *S*. *rhododendrifolia* in the ML tree (Figs. 2, S3 and S4) or *S. rhododendrifolia* and *S*. *obscura* in the Astral species tree (Fig. S5). These species are also distributed in HDM. Thus, our findings indicate that in a genus characterized by common hybridization, incomplete lineage sorting and polyploidization, such as *Salix* (Skvortsov, 1999; Argus et al., 2010; Wagner et al., 2020; Sanderson et al., 2023), it is advisable to include a substantial number of species and their accessions in phylogenetic studies to avoid potentially erroneous relationships. Some nonmonophyletic species occur in different infraspecies (e.g., subspecies and varieties), such as *Salix paraplesia, S. shihtsuanensis, S*. *hypoleuca,* and *S*. *fargesii*. This could be attributable to incorrect infraspecific treatments or natural hybridization. Nonmonophyly of some species, including *S. discolor* and *S. eriocephala*, which are capable of hybridizing with several *Salix* species (Argus et al., 2010), highlights that natural hybridization may be responsible for the extensive nonmonophyletic species in our investigation. In addition, we obtained SRA sequence data for 103 *Salix* samples from NCBI but could not authenticate the voucher specimens for species identification. It is possible that the nonmonophyly of certain *Salix* species may result from significant within-species variability or incorrect identification due to challenges posed by high intraspecific variability and hybrid offspring, which are difficult to discern.

Taken together, the extensive nonmonophyly of the species within our phylogenetic trees may be attributed to hybridization, incomplete lineage sorting, polyploidization, incorrect infraspecific treatments and identification problems. Further studies with more evidence are needed to identify the factor or factors accounting for the extensive nonmonophyly found in our study. These facts hinder efforts to propose a reasonable classification system for *Salix* and understand its evolution. Due to these same factors, sectional classification of *Salix* requires molecular evidence through comprehensive sampling of both species and individuals within each species.

### *4.3* *Implications for subgeneric classification of* Salix

Several subgeneric classifications of *Salix* have been proposed. Skvortsov (1999) classified the species of the former Soviet Union and adjacent countries into three subgenera, namely, *Salix*, *Chamaetia* and *Vetrix*. He also noted that the subgenera *Chamaetia* and *Vetrix* share a closer relationship with each other than with the subgenus *Salix*. Dorn (1976) held the opinion that the classification of the subgenera *Chamaetia* and *Vetrix* could only be made conditionally, because these subgenera are not clearly distinguishable. Therefore, he only recognizes two subgenera, *Salix* and *Vetrix*. In contrast, Ohashi (2001) classified Japanese species into six subgenera, namely, *Salix, Protitea, Pleuradenia, Chosenia, Chamaetia* and *Vetrix*. Argus et al. (2010) used morphological analysis to recognize five subgenera for North American willows (*Longifoliae, Protitea, Salix, Chamaetia* and *Vetrix*). For convenience, we choose to use the notion of seven *Salix* subgenera, considering of systems of Ohashi (2001) and Argus et al. (2010), i.e., *Chosenia, Pleuradenia, Protitea,* *Salix,* *Longifoliae, Chamaetia* and *Vetrix*.

Previous molecular phylogenetic studies have consistently identified two main clades of *Salix* using traditional Sanger sequencing of plastid markers (Azuma et al., 2000; Chen et al., 2010; Hardig et al., 2010; Abdollahzadeh et al., 2011; Lauron-Moreau et al., 2015; Wu et al., 2015; Zhang et al., 2018b; Wagner et al., 2021; Gulyaev et al., 2022): clade 1 (subg. *Salix*) and clade 2 (*Chamaetia-Vetrix*, sect. *Triandrae*, *S. cardiophylla, S. arbutifolia*). However, the resolution within these two clades, particularly within the *Chamaetia-Vetrix* clade, has been generally low in plastid sequence-based (including plastome-based) phylogenies. This could be attributed to the highly conserved *Salix* plastomes in terms of genome structure, gene content and sequence similarity (Huang et al., 2017; Wagner et al., 2021). Our results confirm this finding. Our plastome-based phylogenies successfully resolved the two main clades in *Salix*. However, the resolution within the *Chamaetia-Vetrix* clades was extremely low, as indicated by low support values and short branch lengths (Fig. S1). Traditional phylogenetic inference using Sanger sequencing of nuclear markers such as ITS and ETS regions that lack phylogenetic information has also often failed to resolve the aforementioned large clades or lacks strong support (Leskinen and Alstrom-Rapaport, 1999; Hardig et al., 2010; Abdollahzadeh et al., 2011; Lauron-Moreau et al., 2015; Wu et al., 2015). This can be attributed to the short sequence lengths and intraindividual polymorphisms and recombination observed in the nuclear ribosomal ITS and ETS sequences (Feliner and Rosselló, 2007; Nieto Feliner et al., 2004). Our ML tree (Fig. 1, Fig. 2, S3 and S4), which we generated by combining a super matrix of 1449 SCOs, along with the species tree (Figs. 1B and S5), estimated from pre-calculated unrooted gene trees of these SCOs, has definitively resolved and confirmed the existence of two major clades in *Salix* (clade 1 and clade 2). Clade 1 primarily included subg. *Salix* species, whereas clade 2 consisted of four primary subclades, specifically the subgenera *Chosenia* and *Pleuradenia* proposed by Ohashi (2001) and the section *Triandrae*, subg. *Longifoliae,* as suggested by Argus et al. (2010), and the subgenera *Chamaetia* and *Vetrix* (Skvortsov, 1999; Ohashi, 2001; Argus et al., 2010). These relationships could provide valuable insights into the subgeneric classification of *Salix*.

The subg. *Salix* is acknowledged by all *Salix* systems with subgeneric division. The findings of our study and previous research indicate that subg. *Salix* delimitation needs to be redefined. Subg. *Salix* should include certain sections of subg. *Salix* s.l., namely, sect. *Teraspermae, Wilsonianae, Pentandrae, Salix, Salicaster, Floridanae,* and *Humboldtianae*. It is also important to include certain subg. *Vetrix* species that have mistakenly been placed in subg. *Chamaetia* and *Vetrix*. For example, our plastome and SCO trees consistently grouped *S. shihtsuanensis* and its four other varieties in clade 1, which is consistent with findings from a previous study (Gulyaev et al., 2022). We agree with the explanation provided by Gulyaev et al. (2022) that this species was misclassified in section *Sieboldianae* (subg. *Vetrix*). It is clearly a member of subg. *Salix* based on morphological characters (Fang et al., 1999). Additionally, several groups that previous studies have erroneously placed in subg. *Salix* should be removed (Chen et al., 2010; Wu et al., 2015), i.e., sect. *Triandrae* and *S. cardiophylla* (subg. *Pleuradenia*), and *S. arbutifolia* (subg. *Chosenia*) and several other species, including but not limited to *S. bangongensis, S*. *qinghaiensis, S. sericocarpa,* and *S. sphaeronymphe*.

We identified two species within subg. *Longifoliae* (*Salix exigua* and *S*. *interior*) that form a monophyletic clade. The plastome-based tree indicates that this clade is nested within subg. *Salix.* However, in the SCO-based tree, subg. *Longifoliae* clustered in clade 1 and was a sister to clade 3 (the *Chamaetia-Vetrix* clade). The incongruous placement of *Longifoliae* in the plastid and nuclear trees may be attributed to ancient hybridization as indicated by a previous study (Sanderson et al., 2023). Our results are consistent with those of a previous study in which SNP data were obtained through whole-genome resequencing (Gulyaev et al., 2022). Another study by Lauron-Moreau et al. (2015) examined all eight species of subg. *Longifoliae* and found that they were grouped in clade 1 in both the plastid-based tree and the nuclear ITS tree. However, branch support values were not provided in the ITS tree, making it difficult to identify any phylogenetic tree conflicts. Members of subg. *Longifoliae* exhibit morphological characteristics resembling those of *S. arbutifolia* and certain species of subg. *Chamaetia.* Typically, species within subg. *Longifoliae* exhibit long, narrow leaves and male flowers with two stamens. Furthermore, *S. exigua* and *S. interior* do not exhibit any pollination barrier with subg. *Vetrix* species but show one with subg. *Salix* (Argus et al., 2010). These findings imply a closer relationship between subg. *Longifoliae* and subg. *Vetrix* than between subg. *Longifoliae* and subg. *Salix*. Therefore, we support the treatment of Argus et al. (2010) to exclude subg. *Longifoliae* from subg. *Salix* and treat it as an independent subgenus.

Kimura (1988) established the subgenus *Pleuradenia*, which consists of *Salix* *arbutifolia* and *S. cardiophylla*. Ohashi (2000) classified this subgenus as *Urbaniana*, which was subsequently divided into two subgenera (Ohashi, 2001): *Chosenia* and *Pleuradenia*. Each subgenus contains only one species. According to our SCO-based phylogenies, these two subgenera form a monophyletic clade and are sister to sect. *Triandrae*. *S. arbutifolia* and *S. cardiophylla* are tall trees, reaching 20–30 m in height, which is not common in *Salix*. Both species share similar traits, including pendulous catkins during flowering, dimorphic bracts between males and females, deciduous stigmas, and 5–10 stamens (Ohashi, 2001). In contrast, members of sect. *Triandrae* differ from other *Salix* species in several aspects, such as the bark exfoliating in irregularly shaped patches (Skvortsov, 1999). *Salix triandra* exhibits a low level of glycosides and tannins (Buchler, 1996), possesses the smallest amount of nuclear DNA (Thibault, 1998), and is the only *Salix* species identified to date that hosts a unique rust species (Pei et al., 1999). An AFLP study of the genetic relationships of *Salix* indicated that the genetic similarity between *S. triandra* and subg. *Salix* and subg. *Vetrix* was comparable and greater than the genetic similarity between these subgenera, which might justify the establishment of sect. *Triandrae* as a distinct subgenus (Argus et al., 2010). We agree with Kimura and Ohashi’s proposal to merge the subgenera *Chosenia* and *Pleuradenia* into a distinct subgenus, i.e., subg. *Urbaniana*. Additionally, we support the proposal of Wu et al. (2015) that sect. *Triandrae* should be treated as a separate subgenus, namely, subg. *Triandrae*.

Notably, our SCO and plastome-based trees consistently indicate that *Salix* *kochiana* is nested in sect. *Triandrae*, which is consistent with a previous investigation (Wang et al., 2022). The species of sect. *Triandrae* are primarily marked by a stamen number of 3, although it can also be 2, 4 or 5 (Fang et al., 1999). This indicates that stamen number is not a consistent characteristic within this section. Our previous study discovered repeated stamen number reduction in *Salix* (Wu et al., 2015)*.* The clustering of *S. songarica*, which is a member of sect. *Triandrae*, in the subg. *Vetrix* clade identified in our study and other studies (Chen et al., 2010; Wu et al., 2015) suggests that defining sect. *Triandrae* based on stamen number is unreliable. Although *S. kochiana* shows no morphological similarities to the species of sect. *Triandrae*, our study suggests that there may still be a close affinity between them and that *S. kochiana* could indeed be a member of this section.

The subgenera *Chamaetia* and *Vetrix* have been traditionally classified based on morphological characters, including plant habits (tree, shrub or dwarf shrub), the number of stamens, and the number of nectar glands (Skvortsov, 1999; Argus et al., 2010). However, as the differences are minor and the character states usually overlap, these traits may evolve convergently as adaptations to environmental and climatic conditions (Skvortsov, 1999; Wagner et al., 2018). For example, several important traits used in traditional *Salix* classification, such as dwarf shrubs and connate bud scales, have evolved independently numerous times. In addition, the number of stamens has undergone repeated reductions (Wu et al., 2015; Wagner et al., 2018). These traits are important in *Salix* classification. The boundaries between the subgenera *Chamaetia* and *Vetrix* can be conditionally drawn and are not easily separable. Therefore, Dorn (1976) acknowledges only *Salix* and *Vetrix* as two subgenera*.* However, based on phylogenetic studies from traditional Sanger sequencing and RADseq, Lauron-Moreau et al. (2015) and Wagner et al. (2018), suggest merging *Chamaetia* and *Vetrix* as a single subgenus (i.e., subg. *Verix*). He et al. (2021), Gulyaev et al. (2022), Sanderson et al. (2023) and Marinček et al. (2024) have all resolved the *Chamaetia-Vetrix* clade into a robust monophyletic clade based on RADseq, SNP and 787 genes, respectively. Our phylogenetic results support the treatment of Dorn (1976) that merges subg. *Chamaetia* and *Vetrix* into one subgenus, namely *Vetrix.* This is also supported by Chen et al. (2010), Lauron-Moreau et al. (2015) and Wu et al. (2015). The delimitation of subg. *Vetrix* s.l., as indicated by our results, should consist of species from subg. *Chamaetia* and subg. *Vetrix* s.str., and some species that were wrongly placed in subg. *Salix*, which includes but is not limited to *S. bangongensis, S. qinghaiensis, S. sericocarpa,* and *S. sphaeronymphe* (Chen et al., 2010; Wu et al., 2015).

Previous studies based on a large number of nuclear DNA markers have addressed relationships within the *Chamaetiae-Vetrix* clade, which is the lineage with the most species (approximately 423 species according to our count; Table S10). These studies include Wagner et al. (2018), He et al. (2021), Gulyaev et al. (2022), Sanderson et al. (2023) and Marinček et al. (2024) based on sequence data from RADseq, SNP, 787 genes and RADseq, with 14, 26, 45, 38 and 73 species of the *Chamaetiae-Vetrix* clade sampled, respectively. When we compared our results, which sample 97 species, to those of previous studies, we found that the topologies and relationships of the *Chamaetiae-Vetrix* clade differed with the number of species sampled. For example, in the study of Wagner et al. (2018) with only 14 European *Salix* species sampled for this clade, *Salix reticulata* is sister to the remaining species of the *Chamaetiae-Vetrix* clade, but this relationship doesn’t exist in the other studies that also sampled this species (He et al., 2021; Sanderson et al., 2023; Marinček et al., 2024). In plastome-based studies (Wagner et al., 2021 and our present study), sister species of *S. reticulata* differ when different species are sampled. Moreover, the two accessions of *S. reticulata* are not monophyletic in our plastome-based ML tree. The study of He et al. (2021), Gulyaev et al. (2022) and our current study all show that the *Chamaetiae-Vetrix* clade consists of two robust clades, but the species consisting of these two species differ when the number of species sampled varies. The study by Marinček et al. (2024) demonstrated that the North American species formed a clade sister to a clade consisting of Eurasian species, although there were some exceptions. However, the majority of their samples were collected from middle to high latitude regions (118 out of 197 samples from regions with latitude high than 40° N), which differs from He et al. (2021), Gulyaev et al. (2022) and our current study. This suggests that a comprehensive sampling strategy, covering the full range and as many species as possible, is crucial to fully elucidate the relationships within the *Chamaetiae-Vetrix* clade.

In summary, our current study is the first phylogenomic study of *Salix* using large number of DNA sites from plastomes and nuclear sequences with at least twice the number of species as previous studies: 124 and 128 species for the SCO and plastome datasets, respectively, whereas previous studies sampled no more than 62 species (Gulyaev et al., 2022). This is also the first phylogenomic study to use samples representing all *Salix* lineages identified in previous studies. We therefore obtained a backbone phylogeny that revealed relationships between the main lineages of *Salix.* The genus *Salix* is comprised of two sister clades: the subg. *Salix* s.str. clade and the other clade, which comprises four clades, namely sect. *Triandrae*, subg. *Chosenia-Pleuradenia*, subg. *Longifoliae* and subg. *Chamaetia-Vetrix*, with the first and latter two clades both forming monophyletic clades sister to each other. We support the division of *Salix* into five subgenera, i.e., *Salix*, *Triandrae*, *Urbanianae*, *Longifoliae* and *Vetrix*. It is noteworthy that although our samples represent all major lineages, the species-level sampling fraction of *Salix* was relatively low (approximately 24.4%). Although the majority of species are clustered in corresponding lineages as shown by previous studies and our current study, some species may be placed in incorrect lineages as revealed by phylogenetic studies. For example, our results indicate that *Salix kochiana*, a species belonging to subg. *Vetrix*, consistently clustered with sect. *Triandrae* in both our plastome and SCO based trees. Additionally, some species of subg. *Salix* (e.g., *S. bangongensis, S. qinghaiensis, S. sericocarpa*, and *S. sphaeronymphe*) consistently clustered in the *Chamaetia-Vetrix* clade. Consequently, although the main lineages of *Salix* are resolved, further studies with more sampling are necessary to define the species included in these lineages. In addition, a comprehensive phylogeny of *Salix* needs not only nearly complete sampling at the species level but also the inclusion of accessions that cover the geographic range of each species, as indicated by extensive nonmonophyly of species in our results. Our study has demonstrated that genome skimming can be used as a cost-effective, efficient, and reliable method to obtain large amount of genomic sequence data for phylogenomic studies, thereby facilitating fully resolving relationships of *Salix*. It is important to note, however, that the SCOs assembled by HybPiper based on genome skimming data may be incomplete and contain assembly errors due to inadequate data. This is also a limitation for other similar pipelines, such as Easy353 (Zhang et al., 2022) and Reads2tree (Dylus et al., 2024). It is therefore recommended that phylogenomic analysis be conducted on the basis of complete and accurate sequence data, whenever feasible. For example, RNA sequencing, whole genome sequencing, and whole genome resequencing with adequate and accurate short-read or long-read sequencing data.

### 4.4 Radiation of the Chamaetia-Vetrix *clade*

The *Chamaetia-Vetrix* clade is the most species-rich lineage in *Salix*, comprising approximately 423 species and accounting for approximately 83.1% of *Salix*. Of these, 226 occur in China, and 103 occur in the HDM according to relevant floras and *Salix* monographs (Rechinger, 1992; Fang et al., 1999; Skvortsov, 1999; Ohashi, 2001; Argus et al., 2010).

The estimated divergence time of the *Chamaetia-Vetrix* clade was approximately 26.15 Ma, and the increase in the diversification rate of *Salix* during the early Oligocene was followed by a slowdown in the late Miocene. Alongside the observation that the *Chamaetia-Vetrix* lineage comprises approximately 83.1% of *Salix* species, it is plausible that the origination of the *Chamaetia-Vetrix* lineage has resulted in the higher diversification rate of *Salix*. Most species of *Salix* favor temperate climate conditions and humid environments, such as natural wetlands, riparian vegetation, and arctic-alpine tundra (Skvortsov, 1999; Argus et al., 2010; Wu et al., 2015). A previous study also confirmed that the species diversity and distribution patterns of *Salix* species are significantly influenced by annual precipitation and mean temperature, i.e., water-energy dynamic equilibrium (Li et al., 2019).

The observed acceleration in the *Salix* diversification rate started in the early Oligocene (∼26 Ma) and decelerated in the early Miocene (∼21 Ma). This indicates that the diversification of *Salix* species favors cooler and temperate climates, as the global climate underwent significant changes of ∼33.7 Ma—a time when a nearly universally equable world rapidly became colder and drier (Pittermann et al., 2012). Additionally, tectonic events in the QTP and European Alpine System during this period may have also contributed to the increase in diversity (Hughes and Atchison, 2015; Wu et al., 2022). In response to increased aridity during the Cenozoic era, plants underwent substantial distributional changes, leading to the formation of novel biomes such as grasslands and cactus-dominated deserts (Pittermann et al., 2012). Notably, North America experienced a marked shift from forested regions to more open habitats, such as mosaic woodlands and savannas, during the late Eocene and Oligocene epochs (Samuels and Hopkins, 2017). The high moisture requirement of *Salix* species suggests that the aforementioned change may have hindered their diversification. This is particularly noteworthy during the epoch when both increased and decreased diversification rates coincided with similar temperatures.

Additionally, the monophyly of subg. *Chamaetia* species with distributions in the QTP and in the HDM suggest that the uplift of the QTP and the associated topological and climate changes arising from orogeny could have played a role in the diversification of *Salix* in this region. Many parts of this region were established in the middle Oligocene, and the ongoing uplift subsequently resulted in complex topography, leading to diverse habitats and climates, particularly in the HDM and adjacent areas. This in turn may have facilitated diversification and speciation (Favre et al., 2015; Chen et al., 2019; Wu et al., 2022). The HDM and its surrounding area, located in the southeastern part of the QTP, have the highest species diversity in the entire QTP region (Favre et al., 2015). This high species diversity is also evident in *Salix*. While only five *Salix* species are found in the northwestern region and platform of the QTP, the HDM and adjacent areas contains a total of 103 *Salix* species, accounting for approximately 26.8% of the *Chamaetia-Vetrix* clade. Reliable fossil records of *Salix* with infructescences were discovered from as early as the late Oligocene and early Miocene in Alaska and the Neogene in Europe, indicating a boreal history for *Salix* in the Northern Hemisphere (Collinson, 1992; Boucher et al., 2003). Therefore, our findings, along with those of a previous study (He et al., 2021), corroborate Sun's (2002) hypothesis that *Salix* originated at high latitudes or in arctic regions and then dispersed southward due to climate cooling. Subsequently, *Salix* underwent radiation on the QTP, particularly in HDM and surrounding areas, which may therefore play an important role in the formation of rich species of *Salix*.

## 5 Conclusion

Our phylogenomic analysis indicated that *Salix* can be divided into two major clades, the first containing species of subg. *Salix* (clade 1) and the second encompassing the remaining *Salix* species (clade 2). Although plastome-based phylogenies provided limited resolution, SCO-based datasets yielded fully resolved phylogenies within clade 2. Based on our robust backbone phylogeny of *Salix* and morphological characters, along with previous studies, we support dividing *Salix* into five subgenera: *Salix, Urbaniana, Triandrae, Longifoliae* and *Vetrix.* There are numerous conflicting relationships within *Salix* between the plastome and SCO-based phylogenies, possibly due to recent or ancient hybridization, incomplete lineage sorting, polyploidization, and identification issues. Further studies are needed to determine the exact explanation. We also observed an acceleration of diversification within certain clades corresponding to tectonic and climatic changes during the Oligocene to Miocene epochs. Our robust phylogeny provides a framework for future investigations into macroevolutionary patterns and processes shaping *Salix* diversity.

## CRediT authorship contribution statement

**Kai-Yun Chen:** Investigation, Data curation, Formal analysis, Writing – review & editing. **Jin-Dan Wang:** Investigation, Data curation, Formal analysis. **Rui-Qi Xiang:** Investigation. **Xue-Dan Yang:** Investigation. **Quan-Zheng Yun:** Formal analysis. **Yuan Huang:** Conceptualization, Funding acquisition, Investigation, Writing – original draft – review & editing. **Hang Sun:** Conceptualization, Writing – review & editing, Supervision. **Jia-Hui Chen:** Conceptualization, Methodology, Formal analysis, Investigation, Validation, Funding acquisition, Writing – original draft – review & editing.

## Declaration of competing interest

The author declares no conflict of interest.
