# Supplementary material for: Backbone phylogeny of Salix based on genome skimming data
Source: Plant Divers. 2024 Sep 12;47(2):178–88. doi: 10.1016/j.pld.2024.09.004 (PMC11963080; doi:10.1016/j.pld.2024.09.004)

Fig. S1. Plastome ML tree.

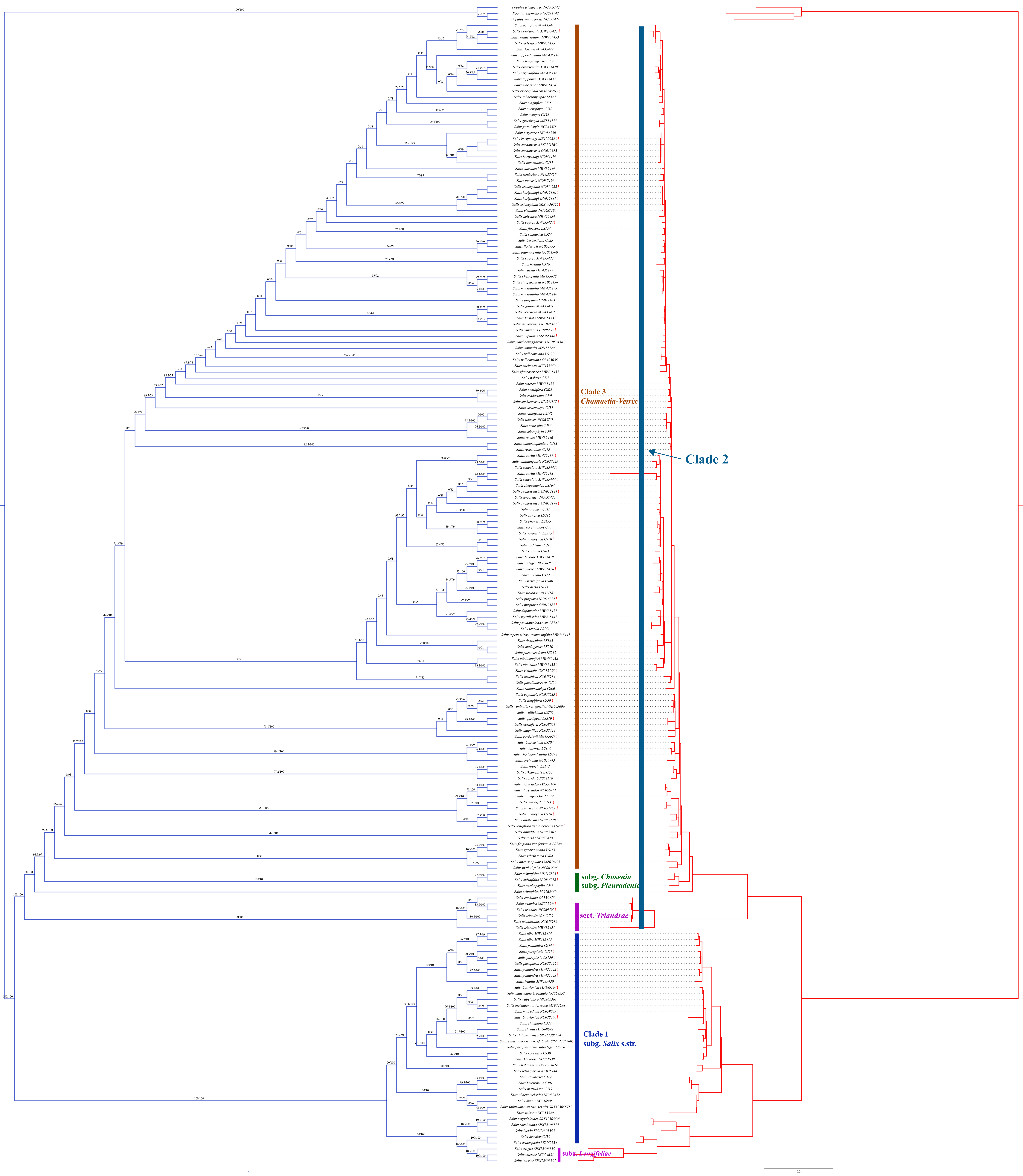

Fig. S2. SCO assembly heatmap.

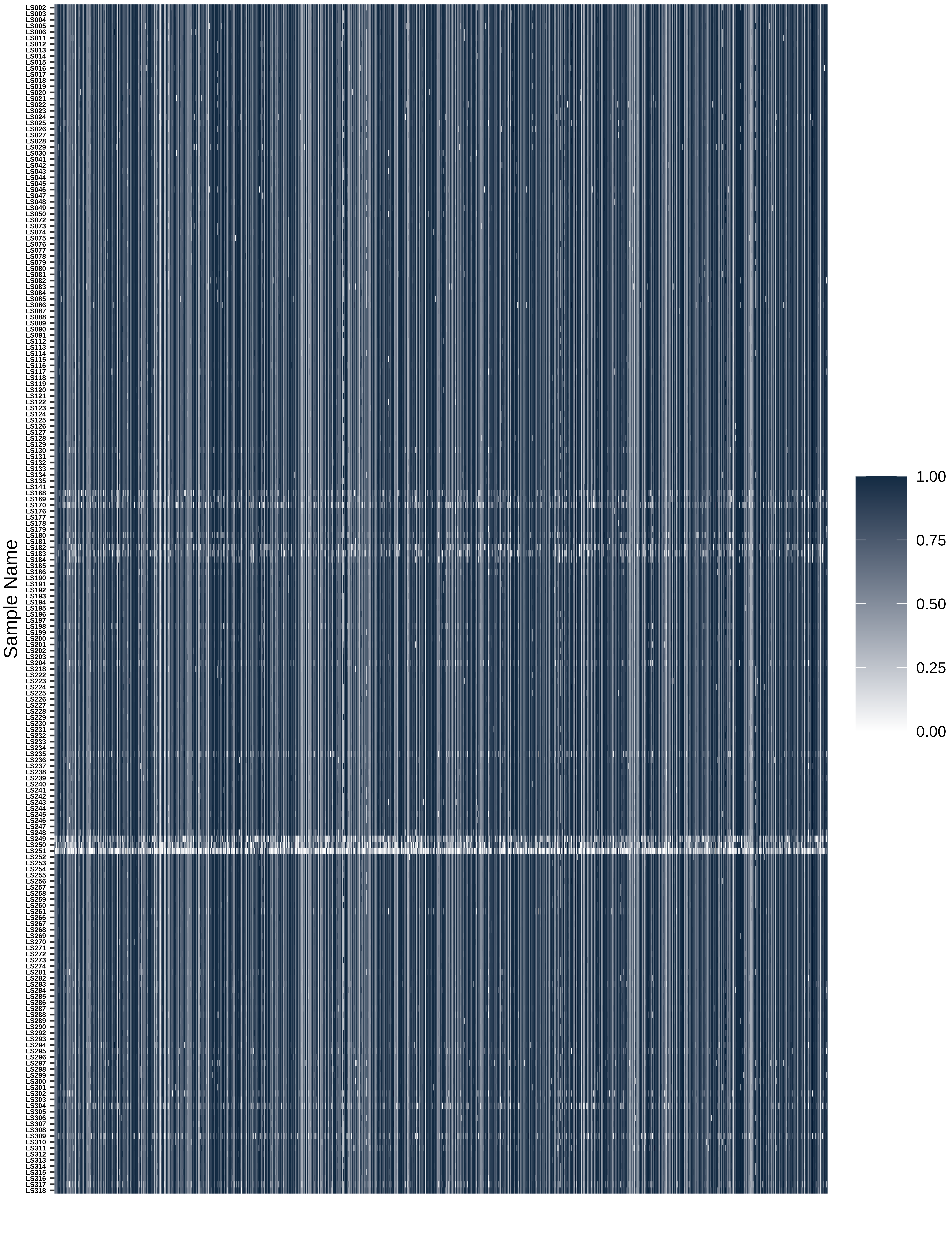

Fig. S3. 1449 SCO ML tree with support value of TBE.

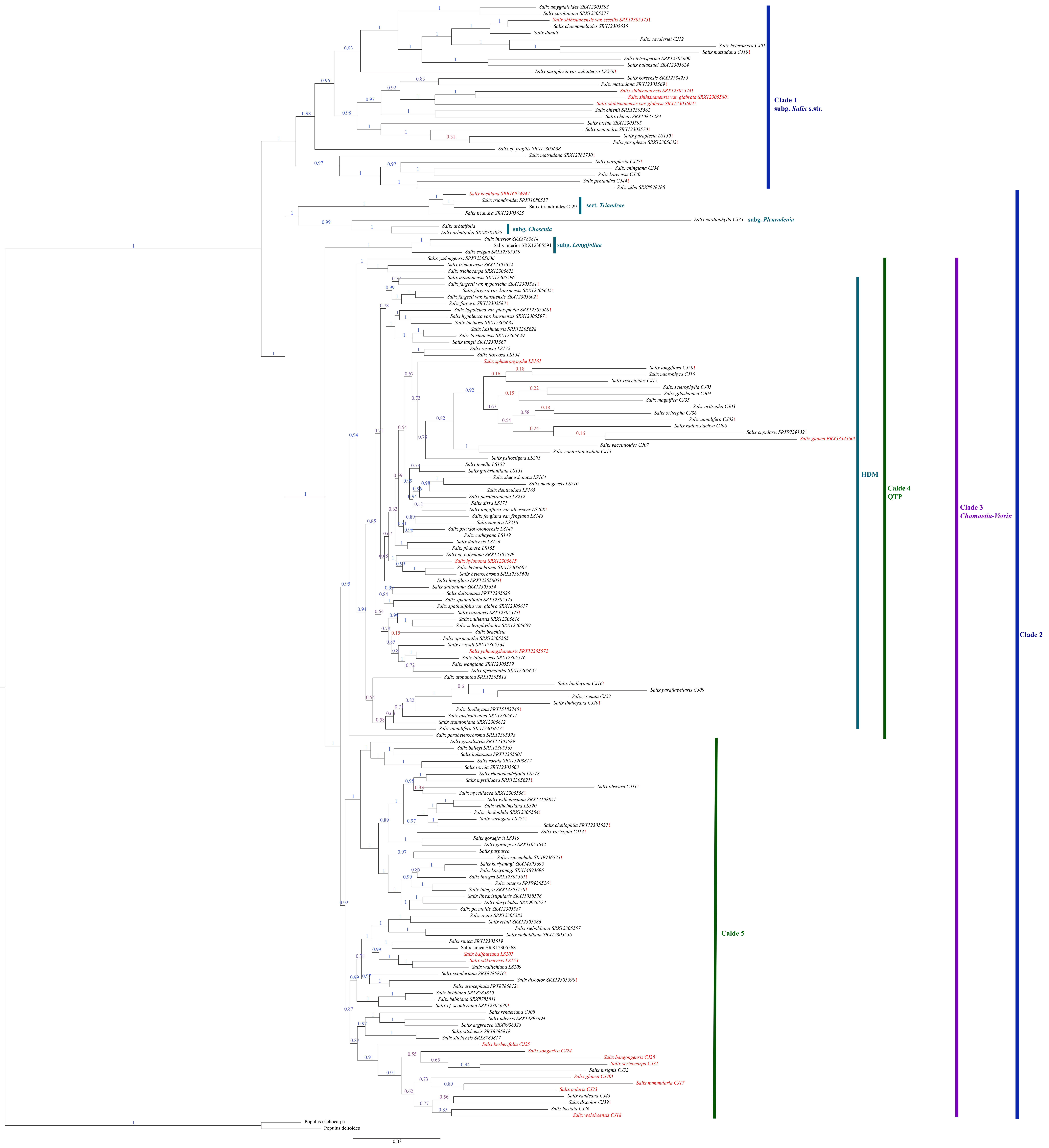

Fig. S4. 1449 SCO ML tree with support value of FBP.

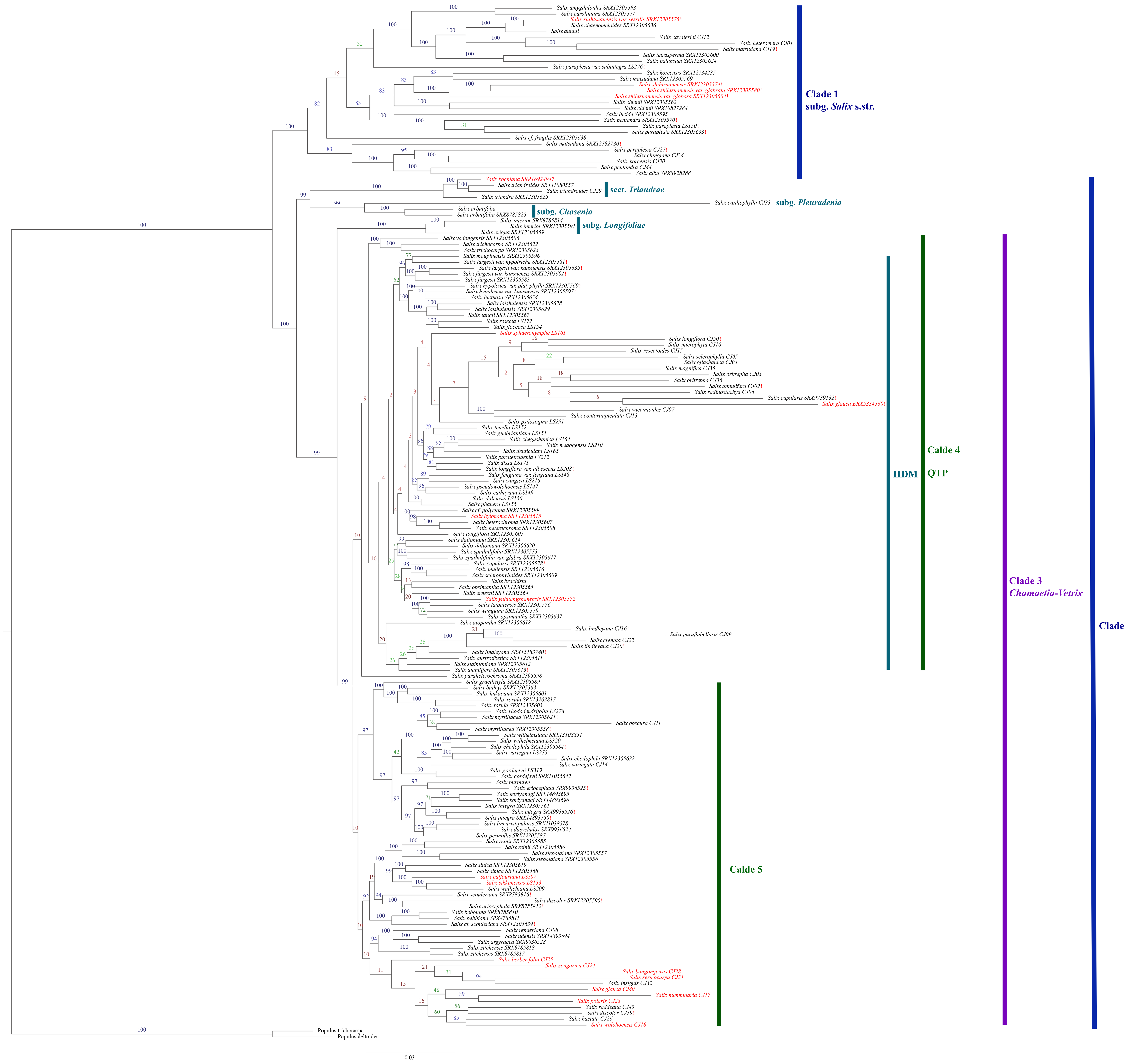

Fig. S5. 1449 SCO Astral species tree.

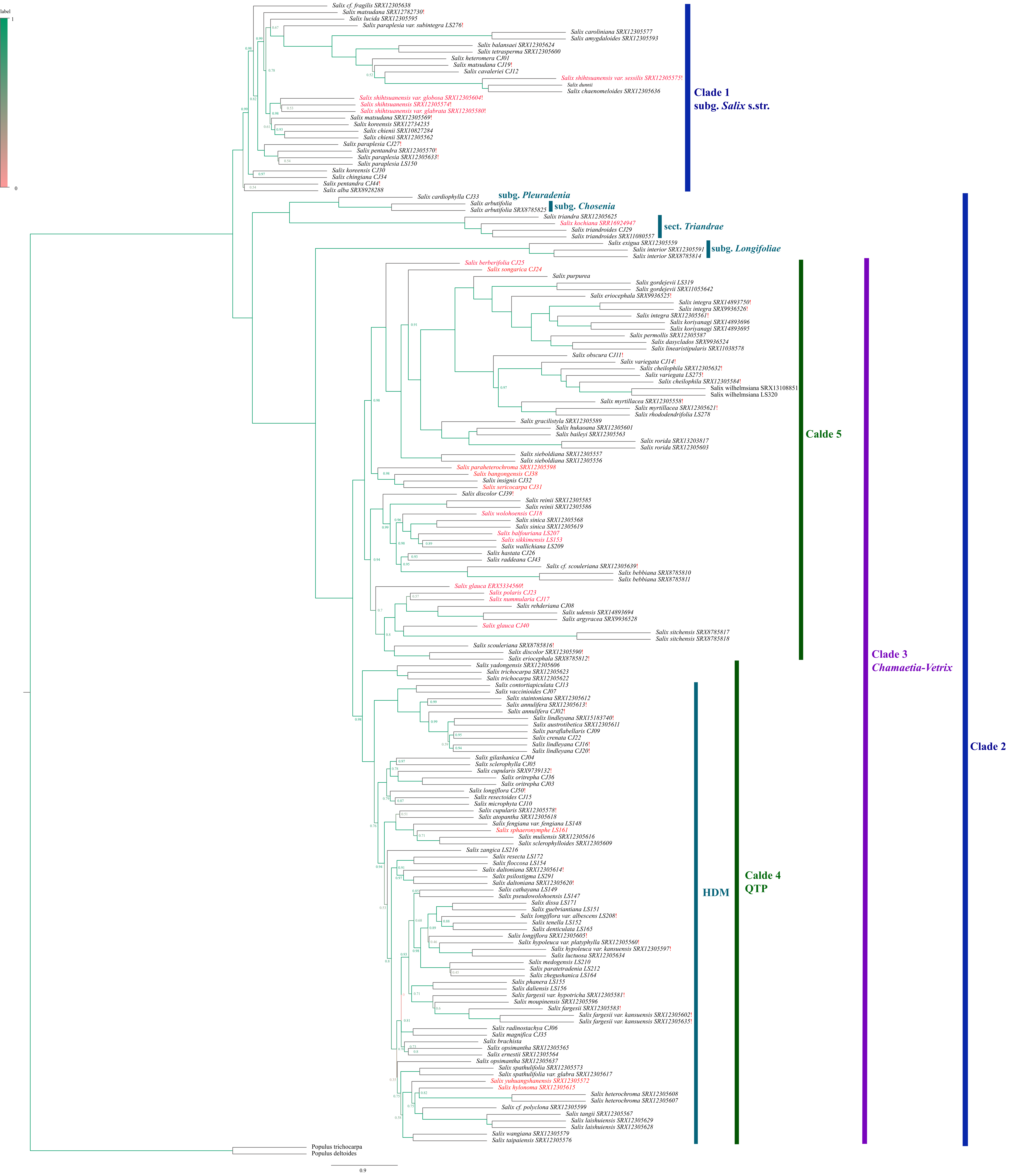

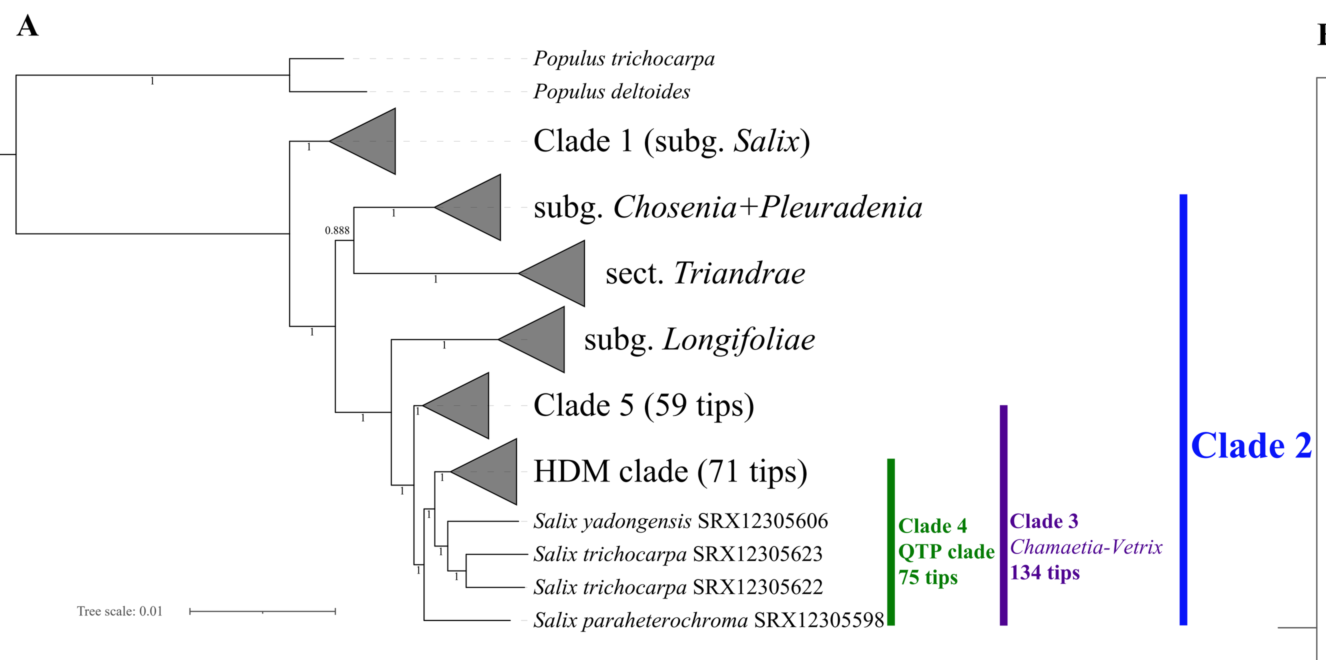

Fig. S6. 1449 SCO Astral species tree.

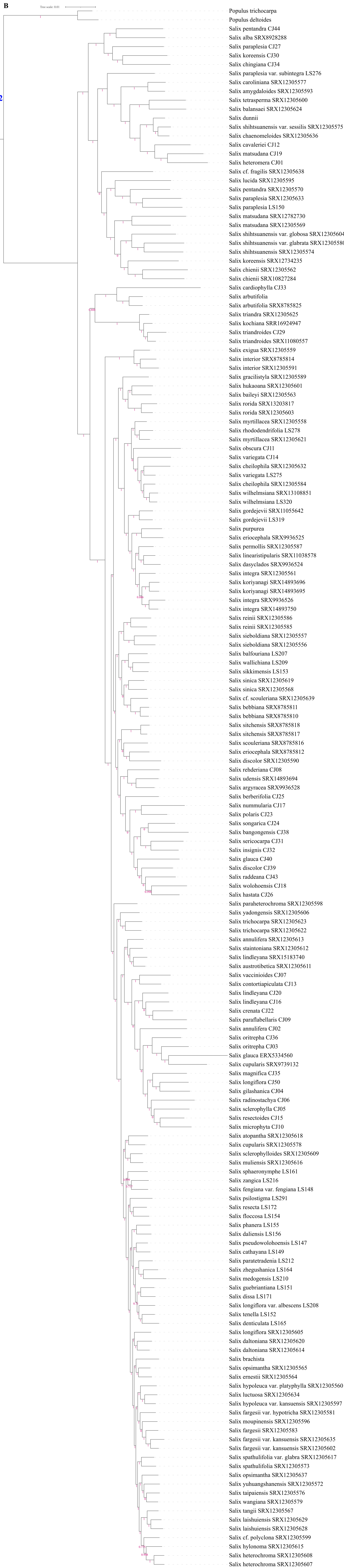

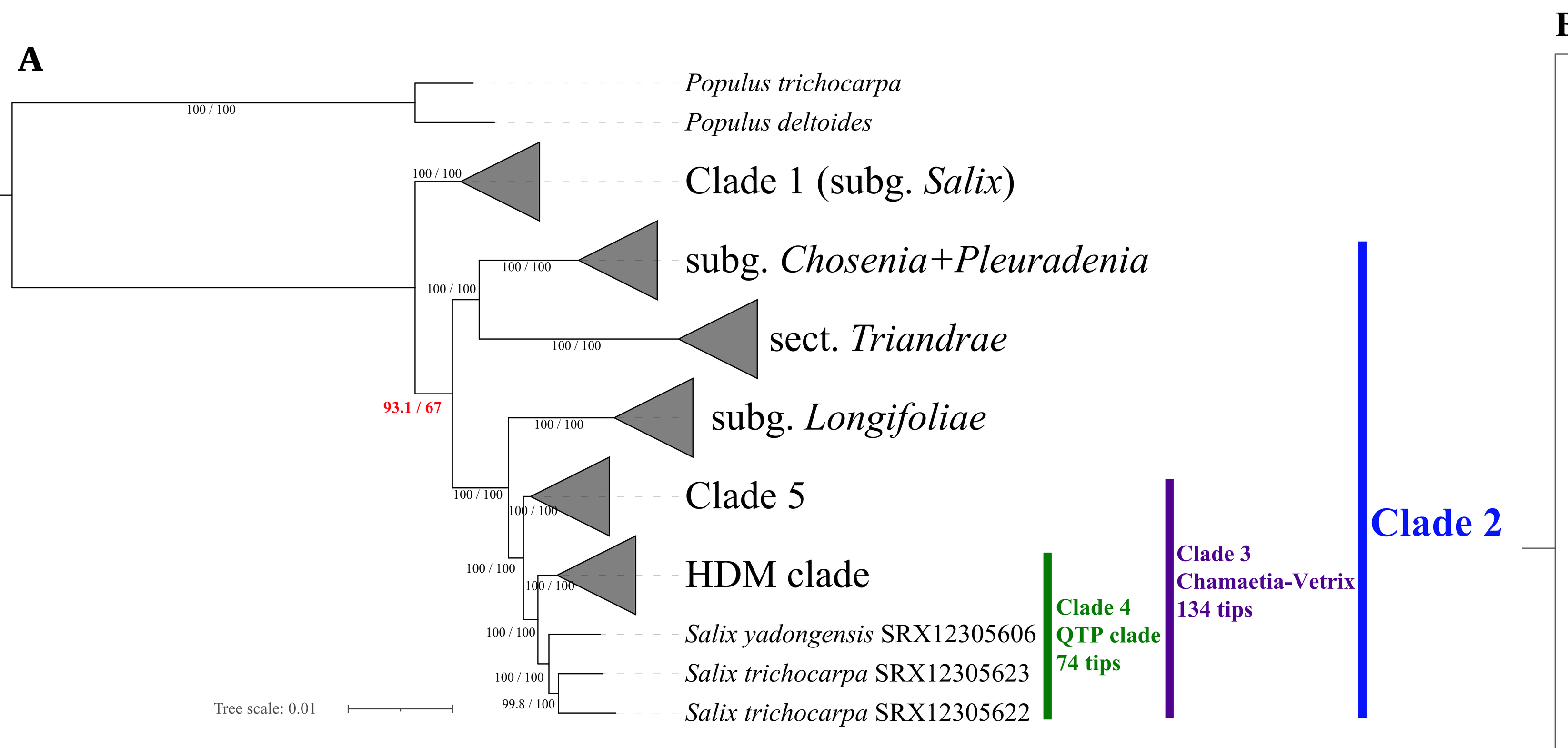

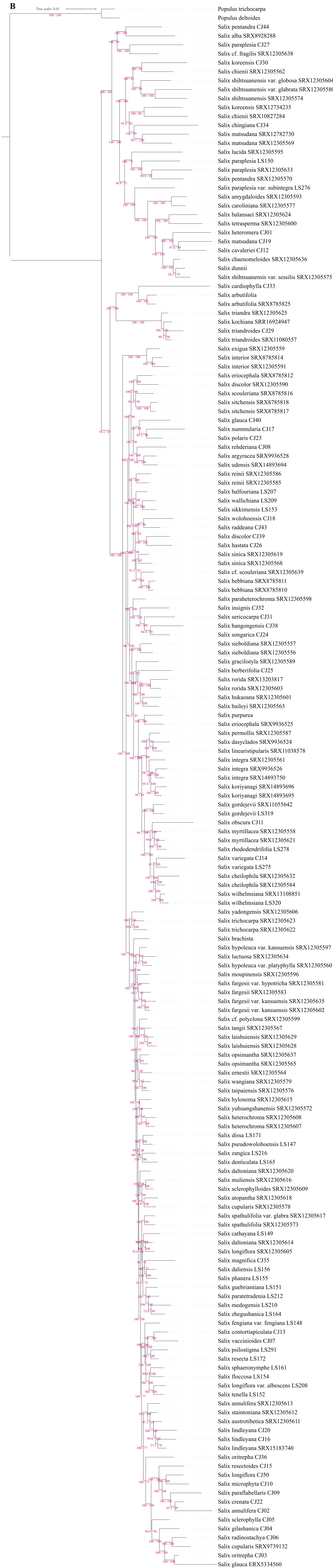

Fig. S7. 253 SCO-11sp ML tree.

Fig. S8. SCO schematic trees.

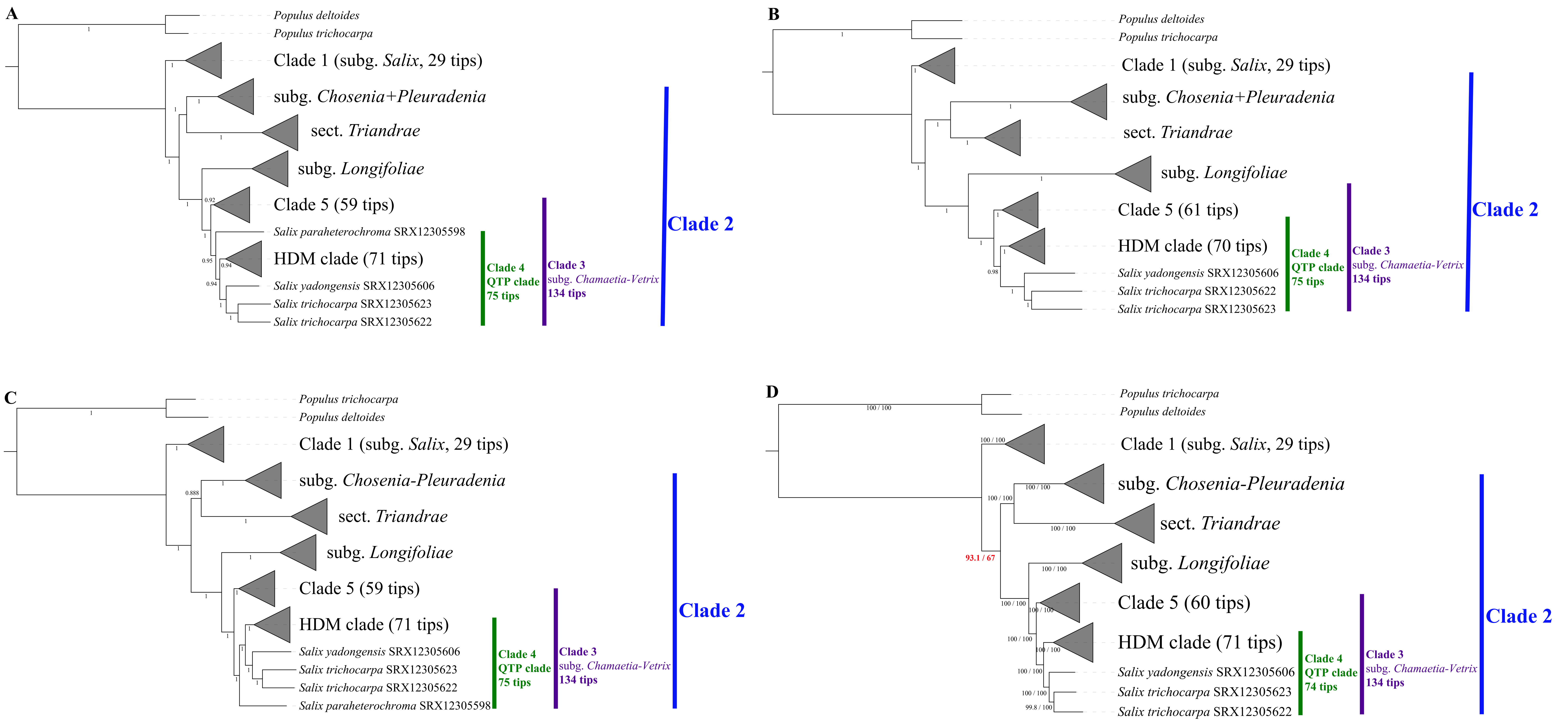

Fig. S9. Chronogram of divergence time estimation.

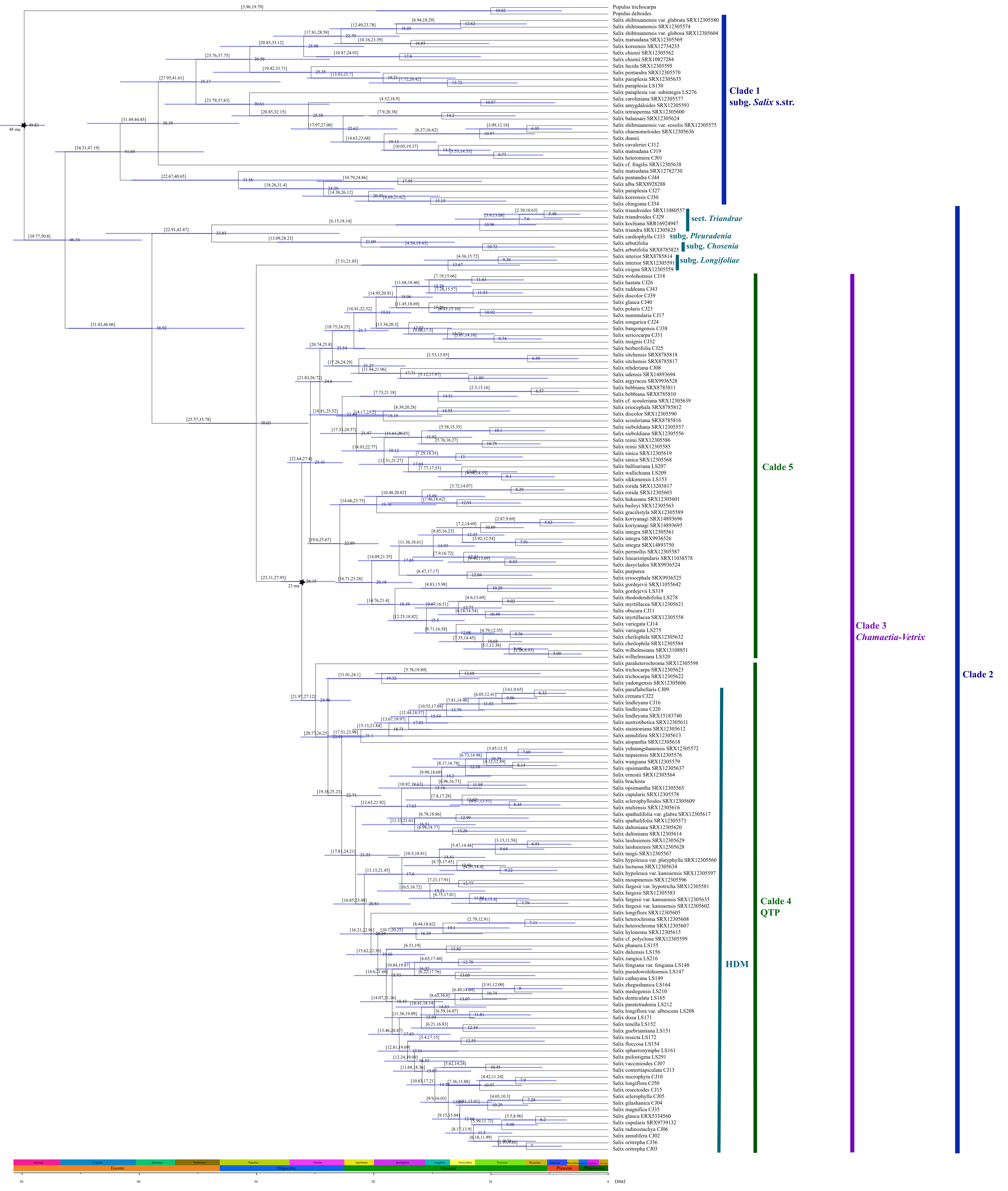

Supplement: Multimedia component 1 [file mmc1.pdf]
