# Supplementary material for: Backbone phylogeny of Salix based on genome skimming data
Source: Plant Divers. 2024 Sep 12;47(2):178–88. doi: 10.1016/j.pld.2024.09.004 (PMC11963080; doi:10.1016/j.pld.2024.09.004)
Supplement: Multimedia component 3 [file mmc3.docx]

**Figure legends:**

Fig. 1. Maximum likelihood tree of *Salix* inferred from the concatenated SCO dataset (A) and the plastome CDS dataset (B). Grey lines connect conflicting taxa between the two phylogenetic trees, and red exclamation points indicate accessions of species that are not monophyletic. The branch supports of the main clades (clades 1, 2, 3) are SHaLRT/UFBoot=100/100 for the palstome-based ML tree and transfer bootstrap expectation (TBE) of 1 for the SCO-based ML tree. Branch support values for all clades are shown detail in Figures S1 and S3.

Fig. 2. Cladogram of concatenation-based species tree inferred by Raxml-ng of 1449 SCOs.

Fig. 3. (A), Net diversification through lineages and the best‐fit rate shift configuration for *Salix*. Colors of branches show the mean diversification rate (species/million years) from BAMM. Red circles indicate node of rate shift with probability. Nodes used for calibration and the corresponding calibrated time are marked with black pentagram. (B), Net diversification rate through time for Salix, and comparison with global climate change over the last 46 million years with the ice-free temperature scale on the right axis and the left δ^18^O temperature scale on the left axis (modified from Zachos et al., 2008).

**Supplementary figure legends:**

Fig. S1. Cladogram (corresponding phylograms are shown in red on the right) of the ML phylogenetic tree of *Salix* was constructed using *Populus* as an outgroup species based on 75 plastid CDSs; branch support values are reported as SHaLRT/UFBoot.

Fig. S2. Heatmaps showing gene recovery efficiency for the 6238 SCOs of Salicaceae. The columns represent genes, and each row represents one sample. Shading indicates the percentage of the reference SCO length cover.

Fig. S3. Concatenation-based species tree inferred by Raxml-ng of 1449 SCOs with GTRGAMMA model. Support values are detected by transfer bootstrap expectation (TBE).

Fig. S4. Concatenation-based species tree inferred by Raxml-ng of 1449 SCOs with GTRGAMMA model. Support values were detected by standard bootstrapping (Felsenstein's bootstrap, FBP).

Fig. S5. ASTRAL‐based species tree of *Salix* based on the 1449 SCO dataset. The numbers above the branches indicate local posterior probability (LPP) values.

Fig. S6. Concatenation-based ML tree inferred by VeryFastTree of 6238 SCO with GTRGAMMA model: (A) schematic tree, (B) detail tree.

Fig. S7. Concatenation-based species tree inferred by IQ-TREE with an edge-linked partition model based on 6238 SCO with GTRGAMMA model: (A) schematic tree, (B) detail tree. Branch support values are reported as SHaLRT/UFBoot.

Fig. S8. Schematic trees for (A) 1449 SCO concatenated ML tree (Fig. S3), (B) 1449 SCO Astral species tree (Fig. S5), (C) ML tree based on 6238 SCOs (Fig. S6), (D) ML tree based on 253 SCO-11sp (Fig. S7).

Fig. S9. Divergence time estimation based on the ML tree constructed from the concatenated 1449 SCO dataset. Nodes used for calibration and the corresponding calibrated time are marked with black pentagram. The numbers below the branches refer to the divergence time (million yeas ago, ma) and its 95% HPD. The blue bars represent the 95% HPD.
